# A Comprehensive Analysis of the Cupin Gene Family in Soybean (*Glycine max*)

**DOI:** 10.1371/journal.pone.0110092

**Published:** 2014-10-31

**Authors:** Xiaobo Wang, Haowei Zhang, Yali Gao, Genlou Sun, Wenming Zhang, Lijuan Qiu

**Affiliations:** 1 School of Agronomy, Anhui Agricultural University, Hefei, China; 2 National Key Facility for Gene Resources and Genetic Improvement/Key Laboratory of Crop Germplasm Utilization, Ministry of Agriculture, Institute of Crop Science, Chinese Academy of Agricultural Science, Beijing, China; 3 Biology Department, Saint Mary's University, Halifax, NS, Canada; East Carolina University, United States of America

## Abstract

Cupin superfamily of proteins, including germin and germin-like proteins (GLPs) from higher plants, is known to play crucial roles in plant development and defense. To date, no systematic analysis has been conducted in soybean (*Glycine max*) incorporating genome organization, gene structure, expression compendium. In this study, 69 putative *Cupin* genes were identified from the whole-genome of soybean, which were non-randomly distributed on 17 of the 20 chromosomes. These Gmcupin proteins were phylogenetically clustered into ten distinct subgroups among which the gene structures were highly conserved. Eighteen pairs (52.2%) of duplicate paralogous genes were preferentially retained in duplicated regions of the soybean genome. The distributions of *GmCupin* genes implied that long segmental duplications contributed significantly to the expansion of the *GmCupin* gene family. According to the RNA-seq data analysis, most of the *Gmcupins* were differentially expressed in tissue-specific expression pattern and the expression of some duplicate genes were partially redundant while others showed functional diversity, suggesting the *Gmcupins* have been retained by substantial subfunctionalization during soybean evolutionary processes. Selective analysis based on single nucleotide polymorphisms (SNPs) in cultivated and wild soybeans revealed sixteen *Gmcupins* had selected site(s), with all SNPs in *Gmcupin10.3* and *Gmcupin07.2* genes were selected sites, which implied these genes may have undergone strong selection effects during soybean domestication. Taken together, our results contribute to the functional characterization of *Gmcupin* genes in soybean.

## Introduction

The cupin superfamily of proteins, mainly consisted of germin and germin-like protein (GLP) subfamilies, is extremely diverse in plants and possess various enzymatic activities such as sugar-binding metal-independent epimerases, and metal-dependent enzymes possessing dioxygenase, and decarboxylase [1], [2]. Germin, initially identified as a specific marker for germination in wheat embryos [3], has been characterized as a homopentameric glycoprotein with oxalate oxidase (OxO) activity [4]. To date, it is speculate to play significant roles in plant development and defense through oxidative breakdown of oxalate, leading to generation of H_2_O_2_ [5], [6]. Germin like proteins (GLPs), with a high sequence and structural similarity to cereal germins, differ from germin as they mostly lack oxalate oxidase activity, and possess activity of SOD and phosphodiesterase [2], [7]–[9]. Cupin-domain has been reported to be associated with the biological properties in plants. For instance, a group of single cupin-domain related proteins, including two phosphomannose isomerases and two epimerases involved in cell wall synthesis, were identified in *Synechocystis PCC6803* genome [10]. Moreover, a duplicated, two cupin-domain GLP protein showed close similarity in structure of an oxalate decarboxylase from the fungus Collybia velutipes and is considered as a putative progenitor of the storage proteins of land plants [10].

Until now, a total of 27 *GLP* genes have been identified in *Arabidopsis*, and their expression vary in different tissues such as roots, leaves and flowers [11]–[13]. Lapik et al reported a cupin-domain protein *AtPirin1* could interact with a CCAAT box binding transcription factor, and served as a downstream component of GPA1 in regulating seed germination and early seedling development [14]. Recently, another two GLP proteins (PDGLP1 and PDGLP2) in *Arabidopsis*, which could interact with *Cucurbita maxima* PHLOEM PROTEIN 16 (Cm-PP16), involved in the regulation of growth of primary root through modulating phloem-mediated resource allocation between the primary and lateral root meristems [15]. The PDGLP1 signal peptide was shown to function in localization to the plasmodesmata (PD) by a novel mechanism involving the endoplasmic reticulum-Golgi secretory pathway. Further, in plum (*Prunus salicina*), two GLP-encoding genes (designated as *Ps-GLP1* and *Ps-GLP2*) were cloned, and the regulation was studied throughout fruit development and during maturity of early and late cultivars. All these demonstrated that GLPs may involve in certain developmental stages in plants.

Expression of Cupin proteins could be modulated by abiotic or biotic stresses, suggesting their multifunctional roles in plant defense response. For instance, a 66-kDa cupin protein BspA (for “boiling-stable protein”), highly expressed in cultured shoots of aspen (*Populus tremula*) in the presence of water stress, was considered to contribute to membrane stability [16]. However, the mechanism of how cupin proteins involve in the plant defense is still not well defined. One germin-like gene (*CchGLP*) cloned from geminivirus-resistant pepper, induced by ethylene and salicylic acid other than jasmonic acid, encoded an enzyme with manganese superoxide dismutase (Mn-SOD)activity [17]. Also, Mn-SOD activity was identified in GLPs isolated from tobacco and Barbula unguiculata [18]–[20]. Considering plant Mn-SODs was distributed extracellularly as well as in mitochondria and peroxisomes and associated with defense against biotic stress in plants [18], [21], [22], it is probably to speculate that Cupin protein may involve in the plant defense through scavenging free radicals.

The ubiquitous distribution of GLPs implies their indispensable and fundamental roles in plants [23], [24]. In soybean, rare studies have been performed on the functional characterization of Cupin proteins [25]. Completion of the soybean genome greatly facilitated the identification of gene families at the whole-genome level [26]. In the present study, a genome-wide identification of Cupin domain was performed in soybean, and detailed analysis of the sequence phylogeny, genome organization, gene structure, expression profiling and selective effects of *Gmcupin* genes during soybean domestication was performed. Our data contributes to the evolutionary and functional analysis of the *Cupin* gene family in soybean.

## Materials and Methods

### Sequence retrieval and phylogenetic analysis

Amino-acid sequence of the Cupin domain was used to search for potential Dof-domain homolog hits in the whole-genome sequence of *Glycine max* with BLASTP at the Phytozome database (http:/www.phytozome.net) [27]. All non-redundant hits with expected values of <1E-5 were collected. Subsequently, manual analysis was performed to confirm the presence of Cupin domain using InterProScan program (http://www.ebi.ac.uk/Tools/InterProScan/) [28].

Sequence alignments of the full-length protein sequences were performed using Clustal X software (version 1.8) [29]. Phylogenetic trees were constructed with MEGA 5.0 using Neighbor-Joining (NJ) method with 1000 replicates of bootstrap analysis [30]. The evolutionary distances were computed using the p-distance method. WebLogo was used to create the distribution of amino-acid residues at the corresponding positions in domain profiles for the conserved Cupin domain of Gmcupins [28].

## Identification of conserved motifs

For the motif analysis, deduced amino-acid sequences of the Gmcupins were analysis by Multiple EM for Motif Elicitation version 4.9.1 (http://meme.nbcr.net/meme/cgi-bin/meme.cgi) [31]. Structural motif annotation was performed using the Pfam (http://pfam.sanger.ac.uk), NCBI (http://www.ncbi.nlm.nih.gov/) and SMART (http://smart.embl-heidelberg.de) database.

## Genomic structure and chromosomal location of *Gmcupins*

The exon/intron organization for individual cupin gene was illustrated with Gene structure display server program (GSDS) (http://gsds.cbi.pku.edu.cn/) [32] by comparing the cDNAs with their corresponding gDNA sequences in the Phytozome database (http://www.phytozome.net/gmax). The chromosomal locations of soybean cupins were mapped to the duplicated blocks using the Chromosome Visualization Tool (CViT) genome search and synteny viewer at the Legume Information System (http://comparative-legumes.org/) [33], [34].

## Calculation of Ka/Ks values

Clustal X (version 1.8) was used for the pairwise alignments of the paralogous nucleotide sequences [29]. Ka (non-synonymous substitution rate) and Ks (synonymous substitution rate) were estimated using the DnaSp v5 program [35]. Divergence time (T) was calculated using as the formula: T = Ks/2λ, where the synonymous mutation rate λ was 6.1×10^−9^ for soybean [26], [36], [37].

## Expression analysis of *GmCupin* genes

Genome-wide transcriptome data of seeds during various developmental stages were downloaded from Soybase database (http://soybase.org/). The transcript data were obtained from vegetative tissues (e.g. young leaf, root and nodule), seed of seven developmental stages (10, 14, 21, 25, 28, 35, and 42 days after flowering), and reproductive tissues (e.g. flower, one cm pod, pod shell of 10 and 14 days after flowering). All transcript data were analyzed with Cluster 3.0 [38] and the heat map was viewed in Java Treeview [39].

## Evolutionary analysis of *Gmcupin* genes

Single nucleotide polymorphisms (SNPs) of the *Gmcupin* genes were downloaded from the SoyKB database (http://soykb.org/) based on the resequencing of wild and cultivated soybean genomes [40]. The ratio of each SNP in wild and cultivated soybean populations was analyzed respectively. The SNP site with reverse distribution ratio in different types of soybean population was defined as a putative selective site throughout domestication.

## Results and Discussion

### Identification of *Cupin* gene family in soybean

In order to identify the *Cupin* gene family in soybean genome, BLASTP was performed against the *G. max* v1.1 genome using the conserved Cupin domain. Afterwards, the obtained sequences were used as secondary queries. A total of 69 non-redundant *Cupin* genes were identified in the soybean genome (Table 1). To identify the conserved Cupin domain, all candidates were subjected to functional analysis using InterproScan program (http://www.ebi.ac.uk/Tools/InterProScan/). Soybean *Cupin* genes were numbered from *Gmcupin01.1* to *Gmcupin 20.4* according to their localization on chromosomes. Peptides consisted of 125–495 (average 224) amino acids were encoded by the identified Gmcupin genes in soybean.

**Table 1:** Summary of Cupin family members in soybean.

Gene Symbol	Gene Locus	Primary transcript	Gene location	Amino Acids	Extrons
Gmcupin01.1	Glyma01g04450	Glyma01g04450.1	Gm01:3990477-3991986	220	1
Gmcupin02.1	Glyma02g01085	Glyma02g01085.1	Gm02:816290-817482	147	2
Gmcupin02.2	Glyma02g03100	Glyma02g03100.1	Gm02:2414696-2415880	220	1
Gmcupin02.3	Glyma02g05010	Glyma02g05010.1	Gm02:4077995-4078612	205	1
Gmcupin03.1	Glyma03g32030	Glyma03g32030.1 Gm03: 39840052 - 39842763		495	4
Gmcupin03.2	Glyma03g38630	Glyma03g38630.1	Gm03:44934345-44933730	218	2
Gmcupin04.1	Glyma04g39040	Glyma04g39040.2	Gm04:45306333-45307231	199	3
Gmcupin05.1	Glyma05g25620	Glyma05g25620.1	Gm05:31685194-31686123	215	2
Gmcupin06.1	Glyma06g15930	Glyma06g15930.1	Gm06:12516557-12517688	228	1
Gmcupin07.1	Glyma07g04310	Glyma07g04310.1	Gm07:3163391-3164534	209	1
Gmcupin07.2	Glyma07g04320	Glyma07g04320.1	Gm07:3167203-3168078	208	1
Gmcupin07.3	Glyma07g04330	Glyma07g04330.1	Gm07:3173276-3174394	208	1
Gmcupin07.4	Glyma07g04340	Glyma07g04340.1	Gm07:3179749-3180833	225	1
Gmcupin07.5	Glyma07g04400	Glyma07g04400.1	Gm07:3202414-3203532	208	1
Gmcupin08.1	Glyma08g08600	Glyma08g08600.1	Gm08:6134739-6134464	215	2
Gmcupin08.2	Glyma08g24320	Glyma08g24320.1	Gm08:18508831-18509842	211	1
Gmcupin09.1	Glyma09g03010	Glyma09g03010.1	Gm09:2110529-2109847	217	2
Gmcupin09.2	Glyma09g08030	Glyma09g08030.1	Gm09:7066672-7066667	135	1
Gmcupin10.1	Glyma10g08360	Glyma10g08360.1	Gm10:7201264-7200871	226	2
Gmcupin10.2	Glyma10g11935	Glyma10g11935.1	Gm10:12509357-12509734	125	1
Gmcupin10.3	Glyma10g28010	Glyma10g28010.1	Gm10:36807794-36807686	221	2
Gmcupin10.4	Glyma10g28020	Glyma10g28020.1	Gm10:36812065-36811696	220	2
Gmcupin10.5	Glyma10g28190	Glyma10g28190.1	Gm10:36981942-36980791	223	2
Gmcupin10.6	Glyma10g31200	Glyma10g31200.2	Gm10:39762112-39761524	198	3
Gmcupin10.7	Glyma10g31210	Glyma10g31210.1	Gm10:39768393-39768098	232	2
Gmcupin10.8	Glyma10g42611	Glyma10g42611.1	Gm10:49519941-49520574	177	3
Gmcupin12.1	Glyma12g09630	Glyma12g09630.2	Gm12:7391558-7392181	207	1
Gmcupin12.2	Glyma12g09640	Glyma12g09640.2	Gm12:7398088-7398957	212	2
Gmcupin12.3	Glyma12g09760	Glyma12g09760.2	Gm12:7531728-7532351	207	1
Gmcupin12.4	Glyma12g31110	Glyma12g31110.1	Gm12:34711894-34712517	207	1
Gmcupin13.1	Glyma13g16960	Glyma13g16960.2	Gm13:20815056-20816399	199	2
Gmcupin13.2	Glyma13g18450	Glyma13g18450.2 Gm13: 22109247 - 22113254		226	4
Gmcupin13.3	Glyma13g22050	Glyma13g22050.1	Gm13:25624544-25624397	239	2
Gmcupin13.4	Glyma13g40360	Glyma13g40360.1	Gm13:40856942-40859117	483	5
Gmcupin15.1	Glyma15g05040	Glyma15g05040.2 Gm15: 3611464 - 3613757		351	7
Gmcupin15.2	Glyma15g13960	Glyma15g13960.1	Gm15:10534152-10533514	215	2
Gmcupin15.3	Glyma15g19510	Glyma15g19510.1	Gm15:16833879-16835243	213	1
Gmcupin15.4	Glyma15g35130	Glyma15g35130.1	Gm15:39672342-39673419	231	1
Gmcupin16.1	Glyma16g00980	Glyma16g00980.1	Gm16:652712-653968	209	1
Gmcupin16.2	Glyma16g00990	Glyma16g00990.1	Gm16:656546-657171	181	2
Gmcupin16.3	Glyma16g01000	Glyma16g01000.1	Gm16:660570-661190	206	1
Gmcupin16.4	Glyma16g06500	Glyma16g06500.1	Gm16:5853241-5853122	221	2
Gmcupin16.5	Glyma16g06520	Glyma16g06520.1	Gm16:5858235-5858104	221	2
Gmcupin16.6	Glyma16g06530	Glyma16g06530.1	Gm16:5860996-5862104	220	2
Gmcupin16.7	Glyma16g06630	Glyma16g06630.1	Gm16:5947078-5946961	221	2
Gmcupin16.8	Glyma16g06640	Glyma16g06640.1	Gm16:5951136-5952363	215	2
Gmcupin16.9	Glyma16g07550	Glyma16g07550.1	Gm16:6838751-6839383	210	1
Gmcupin16.10	Glyma16g07560	Glyma16g07560.2	Gm16:6844140-6843743	188	1
Gmcupin16.11	Glyma16g07580	Glyma16g07580.1	Gm16:6860067-6860840	214	1
Gmcupin17.1	Glyma17g05760	Glyma17g05760.1	Gm17:4052453-4053577	208	1
Gmcupin19.1	Glyma19g09370	Glyma19g09370.2	Gm19:11189789-11189486	181	3
Gmcupin19.2	Glyma19g09810	Glyma19g09810.1	Gm19:11530120-11531183	221	2
Gmcupin19.3	Glyma19g09830	Glyma19g09830.1	Gm19:11554699-11555709	221	2
Gmcupin19.4	Glyma19g09840	Glyma19g09840.1	Gm19:11601504-11602281	221	2
Gmcupin19.5	Glyma19g09860	Glyma19g09860.1	Gm19:11630024-11631147	221	2
Gmcupin19.6	Glyma19g09990	Glyma19g09990.1	Gm19:11922733-11922620	221	2
Gmcupin19.7	Glyma19g24840	Glyma19g24840.1	Gm19:30509520-30510472	212	2
Gmcupin19.8	Glyma19g24850	Glyma19g24850.1	Gm19:30514099-30513955	221	2
Gmcupin19.9	Glyma19g24870	Glyma19g24870.2	Gm19:30532519-30533617	221	2
Gmcupin19.10	Glyma19g24900	Glyma19g24900.1	Gm19:30559228-30560036	221	2
Gmcupin19.11	Glyma19g24910	Glyma19g24910.1	Gm19:30576453-30577383	219	2
Gmcupin19.12	Glyma19g27580	Glyma19g27580.1	Gm19:34882234-34884637	212	2
Gmcupin19.13	Glyma19g34780	Glyma19g34780.1 Gm19: 42366324 - 42369290		481	4
Gmcupin19.14	Glyma19g41070	Glyma19g41070.2	Gm19:47390045 - 47391131	184	3
Gmcupin19.15	Glyma19g41220	Glyma19g41220.1	Gm19:47524955-47524382	219	2
Gmcupin20.1	Glyma20g22180	Glyma20g22180.1	Gm20:32106832-32105935	224	2
Gmcupin20.2	Glyma20g25430	Glyma20g25430.1	Gm20:35111115-35111738	207	1
Gmcupin20.3	Glyma20g36300	Glyma20g36300.1	Gm20:44453211-44454264	232	2
Gmcupin20.4	Glyma20g36320	Glyma20g36320.1	Gm20:44458621-44459718	222	2

**Figure 1:**
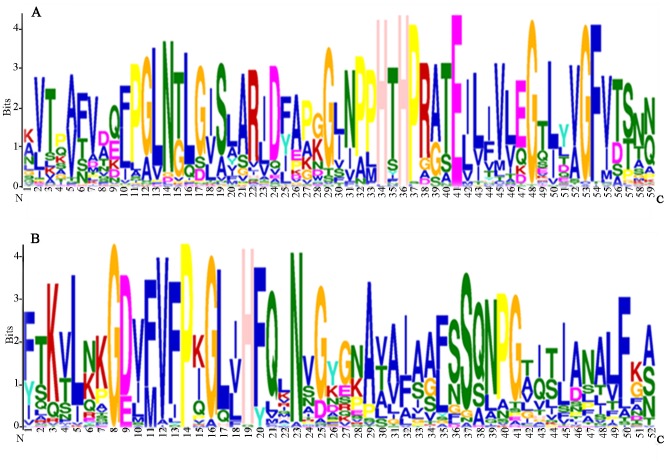
Conserved domains across cupin proteins in soybean. The sequence logos are based on alignments of 69 Gmcupin domains. Multiple alignment analysis of all typical Gmcupin domains (A: Gmcuppin 1; B: Gmcupin 2) were performed with Clustal W. The bit score indicates the information content for each position in the sequence.

Multiple alignment analysis was performed to discover the features of the homologous domain sequence and the frequency of the amino-acids at each position of the Gmcupin domains. Multiple EM for Motif Elicitation was used to identify the putative cupin motif. Two conserved domains, designated as Gmcupin 1 and Gmcupin 2, were found in these Gmcupins, and were formed by 59 amino acids and 52 amino acids, respectively. In Gmcupin 1, seven highly conserved residues were identified, including H-34, H-36, P-37, E-41, Gly-48, Gly-53 and F-54. In Gmcupin 2, four conserved residues were identified such as Gly-8, P-14, H-19 and N-23 (Figure 1). In the previous reports, the histidines and glutamic acid(s) have been reported to act as ligands for the active-site metal [18], [19], [41]. Additionally, studies showed that a set of conserved histidine residues employed in sugar-binding in the ancestral non-enzymatic domain evolved into the metal-coordinating histidine residues in oxalate oxidase [42] and oxalate decarboxylase [43].

### Phylogenetic relationships and gene structure of soybean *Cupin* genes

**Figure 2:**
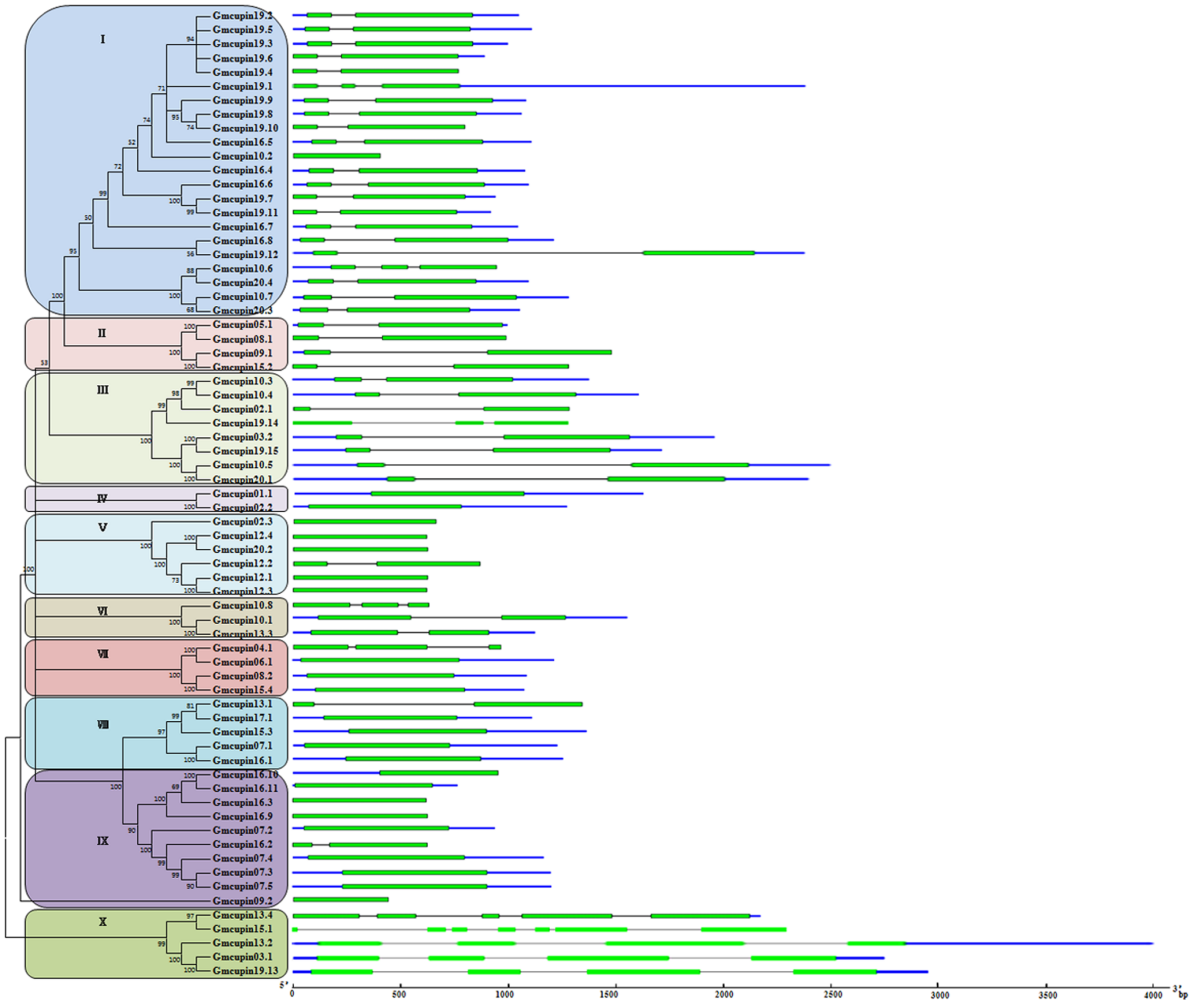
Phylogenetic relationships and gene structure of Gmcupin genes. The phylogenetic tree of Gmcupin proteins constructed from a complete alignment of 69 Gmcupin proteins using MEGA 5.0 by the neighbor-joining method. The bootstrap consensus tree inferred from 1000 replicates is taken to represent the evolutionary history of the taxa analyzed. Percentage bootstrap scores of>50% are indicated on the nodes. Ten major phylogenetic subgroups (designated as I to X) are indicated. Exons of *Gmcupin* genes are represented by green boxes and introns and untranslated region (UTR) by black and blue lines. The sizes of exons and introns can be estimated using the scale below.

The abundance of *Gmcupin* genes may derive from multiple gene duplication events, which was represented by a whole-genome duplication following multiple segmental and tandem duplications [44]. In this study, an unrooted tree was constructed to examine the phylogenetic relationships among the Cupin domains using alignments of the full-length amino-acid sequences in all Gmcupin proteins (Figure 2). The *Gmcupin* gene family was classified into ten subgroups (I-X) with 2-22 members in each subgroup. The very high bootstrap value in each subgroup suggested a common origin for the *Gmcupin* gene in each group except subgroup I. Surprisingly, 12 *Gmcupin* genes (80%) on chromosome 19 were classified into subgroup I with five genes (*Gmcupin19.2*, *Gmcupin19.3*, *Gmcupin19.4*, *Gmcupin19.5* and *Gmcupin19.6*) showed the same base composition. Phylogenetic tree topology revealed that 22 Gmcupin pairs located at the terminal nodes shared high similarities. Thus, they were assigned as paralogous pairs (homologous genes that diverged by gene duplication, Figure 2). These paralogous pairs of *Gmcupin* genes, accounted for more than 63% of the entire Gmcupin family, and showed a sequence similarity of 77.2%∼100% (Table S1). This implied that these genes may evolve from a recent soybean genome duplication event [45].

As gene structural diversity is a possible explanation to the evolution of multigene families, the exon/intron organization in the coding sequences of each *Gmcupin* gene was compared. According to their predicted structures, extremely similar gene structures were observed in most of the closely related *Gmcupin* members within the same. In addition, the position and length of intron were almost completely conserved (Fig. 2A). For instance, most of the *Gmcupin* genes in subgroup I, II and III contained one intron respectively, except *Gmcupin 19.4*, *Gmcupin 10.2*, *Gmcupin 10.6* in subgroup I and *Gmcupin 19.14* in subgroup III. Meanwhile, no introns were identified in 23 of the *Gmcupin* genes (23/30) in subgroup IV, V, VI, VII, VIII and IV, respectively. In contrast, the gene structures in *Gmcupin* subfamily X appeared to be more variable and displayed the largest number of exon/intron structure variants compared with the other *Gmcupin* genes. The dissimilarity of intron phases between subfamilies and the conservation within *Gmcupin* subfamilies may reciprocally support to the results of phylogenetic analysis and genome duplication.

### Chromosomal location and duplication of soybean *Cupin* genes

**Figure 3:**
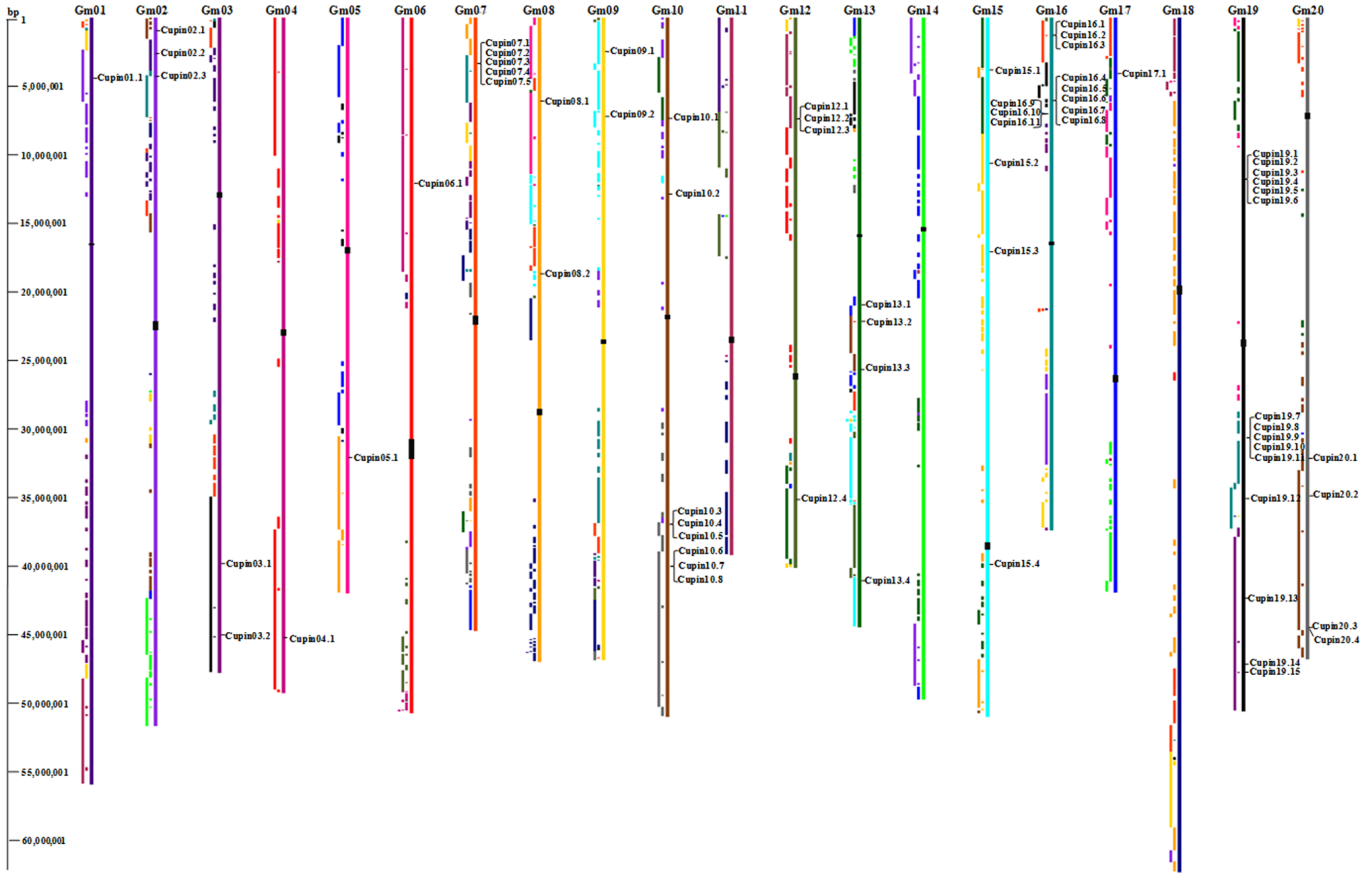
Chromosomal locations and predicted clusters for *Gmcupin* genes. The schematic diagram of genome-wide chromosome organization and segmental duplication arising from the genome duplication event in soybean was derived from the CViT genome search and synteny viewer at the Legume Information System (http://comparative-legumes.org). The chromosomal positions of all *Gmcupin* genes were mapped on each chromosome. Colored blocks to the left of each chromosome show duplications with chromosomes of the same color. The chromosome numbers are indicated at the top of each bar and sizes of chromosomes are represented by the vertical scale. The locations of centromeric repeats are shown as black rectangles over the chromosomes.

As revealed in Figure 3, *Gmcupin* genes were non-randomly distributed on 17 of the 20 chromosomes. Fifteen *Cupin* genes were localized on chromosome 19, while eleveen genes were localized on chromosome 16. In contrast, no more than two *Gmcupins* genes were localized on eleven chromsomes. What's more, no *Cupin* genes were distributed on chromosome 11, 14 and 18, respectively. Most *Gmcupins* presented substantial clustering on several chromosomes especially on those with high densities of the genes. To be exact, 10 *Gmcupin* genes on chromosome 16 were arranged in four clusters, with each in less than 9-kb (*Gmcupin16.1*, *Gmcupin16.2*, and *Gmcupin16.3* located within 8.5-kb; *Gmcupin16.4*, *Gmcupin16.5* and *Gmcupin16.6* located within 8.8-kb; *Gmcupin16.7* and *Gmcupin16.8* located within 5.3-kb; *Gmcupin16.9* and *Gmcupin16.10* located within 5-kb), the other *Gmcupin* gene *Gmcupin16.11* is also close to its neighbor *Gmcupin16.10* within a 1.7-kb segment. Similarly, *Gmcupin19.7* and *Gmcupin19.8* located within a 4.5-kb segment, while *Gmcupin19.10* and *Gmcupin19.11* located within a 19-kb segment on the same chromosome.

Soybean genome is speculated to undergo at least two rounds of genome-wide duplication followed by multiple segmental duplication, tandem duplication, transposition events (e.g. retroposition and replicative transposition) [45]. A tandem duplication event was confirmed by the presence of two or more genes on the same chromosome, while a segmental duplication event was defined as gene duplication on different chromosomes [46]. To our knowledge, the major causes for gene-family expansion were segmental duplication, tandem duplication, and transposition events. To reveal the potential relationship between putative paralogous pairs of *Gmcupin* gens and segmental duplications, CViT genome search and synteny viewer (http://comparative-legumes.org/) were used to map the *Gmcupin* genes [33]. The distributions of *Gmcupin* genes relative to the corresponding duplicate blocks were illustrated in Figure 3. Within the identified duplicated blocks associated with a duplication event, about 18 (81.8%) of *Gmcupins* were preferentially retained duplicates that located in duplicated regions, with 13 putative paralogous pairs located in a segmental duplication of a long fragment (>1 Mb) and 4 located in a segmental duplication of a short fragment (<1 Mb, Table 2). Meanwhile, another putative paralogous pairs (*Gmcupin10.3/Gmcupin10.4*) were formed, which was supposed to be possibly due to tandem duplication in the same orientation. Taken together, we implied that long segmental duplication was predominant for evolution of *Gmcupin* genes, which may be associated with tandem duplication.

**Table 2:** Duplicated Cupin genes in soybean and the dates of the duplication blocks.

Gene1	Gene2	Duplication	Fragment size	Ka	Ks	Ka/Ks	Date(Mya)
Gmcupin5.1	Gmcupin8.1	Fragment	Large	0.0370	0.1177	0.3144	9.65
Gmcupin9.1	Gmcupin15.1	Fragment	Large	0.0328	0.1441	0.2276	11.81
Gmcupin3.1	Gmcupin19.13	Fragment	Large	0.0576	0.1479	0.3895	12.12
Gmcupin10.5	Gmcupin20.1	Fragment	Small	0.0181	0.1073	0.1687	8.80
Gmcupin1.1	Gmcupin2.2	Fragment	Large	0.0161	0.1972	0.0816	16.16
Gmcupin10.1	Gmcupin13.3	Fragment	Large	0.0457	0.1493	0.3061	12.24
Gmcupin13.1	Gmcupin17.1	Fragment	Small	0.1186	0.1838	0.6453	15.07
Gmcupin7.1	Gmcupin16.1	Fragment	Large	0.0173	0.1582	0.1094	12.97
Gmcupin16.2	Gmcupin7.4	Fragment	Large	0.0404	0.1916	0.2109	15.70
Gmcupin4.1	Gmcupin6.1	Fragment	Large	0.1079	0.2469	0.4370	20.24
Gmcupin8.2	Gmcupin15.4	Fragment	Small	0.0290	0.0965	0.3005	7.91
Gmcupin10.7	Gmcupin20.3	Fragment	Large	0.0270	0.1925	0.1403	15.78
Gmcupin10.6	Gmcupin20.4	Fragment	Large	0.0330	0.1511	0.2184	12.39
Gmcupin16.8	Gmcupin19.12	Fragment	Small	0.2215	0.3497	0.6334	28.66
Gmcupin19.13	Gmcupin3.1	Fragment	Large	0.0576	0.1479	0.3895	12.12
Gmcupin13.4	Gmcupin15.1	Fragment	Large	0.0658	0.1742	0.3777	14.28
Gmcupin19.15	Gmcupin3.2	Fragment	Large	0.0239	0.1799	0.1329	14.75
Gmcupin10.3	Gmcupin10.4	Tandem Repeat		0.0351	0.0909	0.3861	7.45

To investigate whether Darwinian positive selection is involved in the divergence of *Gmcupin* genes after duplication and trace the dates of the duplication blocks, the substitution rate ratios (Ka/Ks) of 18 paralogous pairs are calculated using DnaSP program. Ks was used to calculate the approximate dates of duplication events. The segmental duplications of the *Gmcupin* genes in soybean was supposed to originate from 7.45 Mya (million years ago, Ks = 0.0909) to 28.66 Mya (Ks = 0.3497), with a mean value of 13.78 Mya (Ks = 0.1682, Table 2). Meanwhile, the Ks of tandem duplication of *Gmcupin10.3* and *Gmcupin10.4* was 0.0909, dating the duplication event at 7.45 Mya. Considering the fact that the soybean genome underwent two polyploidy events at 13 and 58 Mya, all the segmental duplications of the *Gmcupin* genes occurred around 13 Mya when *Glycine*-specific duplication occurred in the soybean genome [26].

Generally, a Ka/Ks of less than 1 demonstrates a functional constraint with purifying or negative selection of the genes. In this study, The Ka/Ks ratios of 8 segmental duplication pairs were less than 0.3, while the ratios of the other 9 segmental duplication pairs and one tandem duplication pair were more than 0.3, which demonstrated a possibility of significant functional divergence of some *Gmcupin* genes after the duplication events. The Ka/Ks ratios of another two paralogous gene pair (*Gmcupin16.8/19.12* and *Gmcupin13.1/17.1*) were slightly larger than 0.5 (Table 2). This suggests that they experience relatively rapid evolution following duplication. On this basis, we concluded that *Gmcupin* gene family experienced strong purifying selection pressure with limited functional divergence after segmental duplications.

### Differential expression profile of *Gmcupin* genes

**Figure 4:**
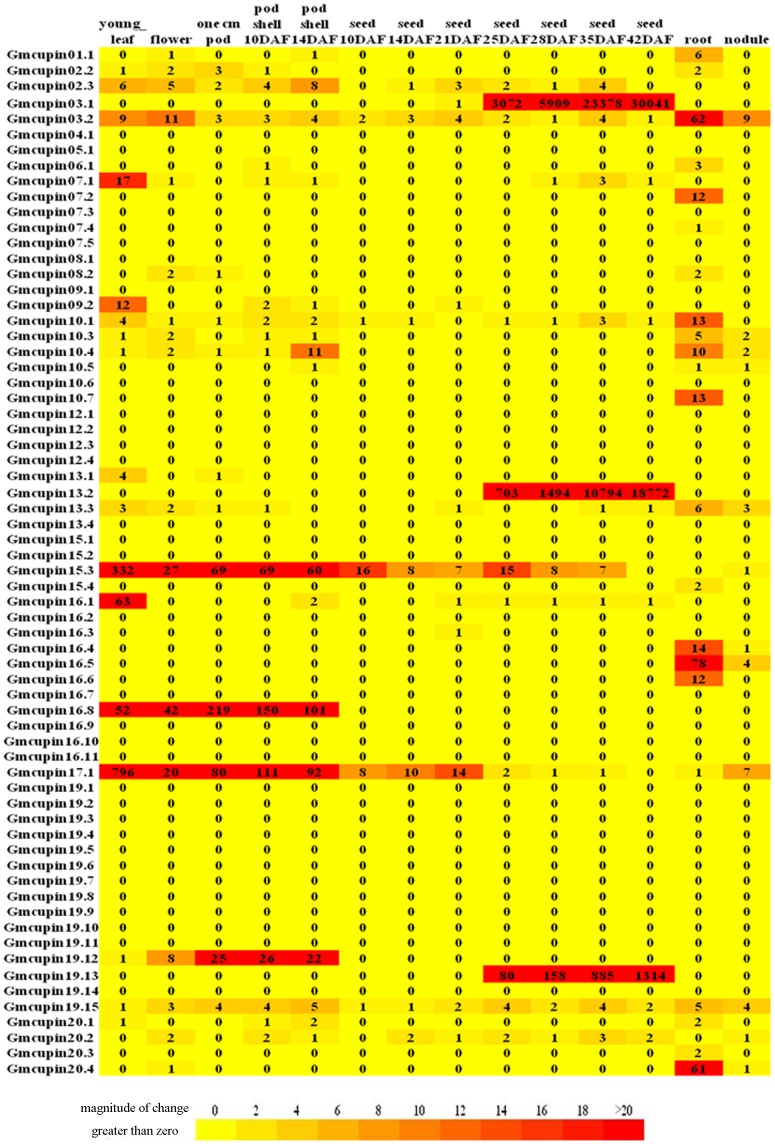
Expression profile of *Gmcupin* genes in different tissues. The numbers in the expression profile are normalized data, which were calculated as reads/kilobase/million normalization of the raw data. All data were downloaded from the SoyBase.

**Figure 5:**
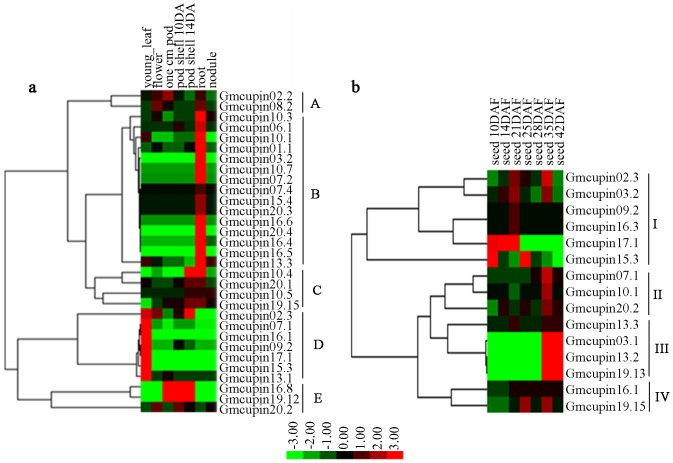
Expression profiles of 35 expressed *Gmcupin* genes in different tissues. a. Heatmap showing hierarchical clustering of 35 expressed *Gmcupin* genes among various tissues analyzed. b. Heatmap showing hierarchical clustering of 35 expressed *Gmcupin* genes during the development of soybean seeds.

To highlight the expression profiles of *Gmcupin* genes, we then analyzed the previously publicly-available RNA-Seq data regarding seven soybean tissues, three pod development stages and seven seed developmental stages. Thirty-five *Gmcupin* genes had sequence reads in at least one tissue, and most of them showed a distinct tissue-specific expression pattern (Figure 4). For example, two genes (*Gmcupin17.1* and *Gmcupin15.3*) had a significantly higher transcript accumulation in the young leaf of soybean. *Gmcupin16.8* was mainly expressed during pod development, while *Gmcupin16.5*, *Gmcupin03.2* and *Gmcupin20.4* were specifically expressed in soybean root. Besides, three genes (*Gmcupin03.1*, *Gmcupin13.2* and *Gmcupin19.13*) of subfamily X were highly expressed at the later stage of seed development. Most *Gmcupin* genes showed a relative low expression level in soybean nodule (Figure 4). These genes were clustered into five groups (A–E) and four groups (I–IV) based on their expression patterns in soybean tissues (excet seeds) and the expression profiles during seven soybean seed development stages (Figure 5). The genes in clusters A–E were mainly expressed in flower/root, root, pod/root, young leaf and pod, respectively. Six genes in cluster I mainly expressed during the early stage of soybean seed development, while seven genes in cluster II and III mainly expressed during the later stage of soybean seed development. In addition, three genes in cluster III having a much higher and specific expression level during soybean seed development from 25 days after flower (DAF) to 42DAF. Further, genes of cluster IV were expressed in most stages of soybean seed development.

The evolutionary fates of duplicate genes may be classified into subfunctionalization (partition of original functions), neofunctionalization (acquisition of novel functions), or nonfunctionalization(loss of original functions) [47]. In this study, we investigated the functional redundancy of *Gmcupin* genes with high proporation of segmental/tandem duplications. Six paralogous pairs (*Gmcupin03.1/19.13*, *Gmcupin07.1/16.1*, *Gmcupin10.1/13.3*, *Gmcupin01.1/02.2*, *Gmcupin16.8/19.12* and *Gmcupin10.7/20.3*) derived from segmental duplications and one paralogous pair (*Gmcupin10.3/10.4*) derived from tandem duplication shared almost identical expression patterns. In contrast, the expression patterns of another seven paralogous pairs (*Gmcupin17.1/13.1*, *Gmcupin08.2/15.4*, *Gmcupin04.1/06.1*, *Gmcupin12.4/20.2*, *Gmcupin10.5/20.1*, *Gmcupin10.6/20.4* and *Gmcupin3.2/19.5*) diversified significantly. These findings indicated that expression profiles of *Gmcupins* have diverged substantially after segmental/tandem duplications. Therefore, we speculate that *Gmcupins* have been retained by substantial subfunctionalization during soybean evolutionary processes.

### Artificial selection analysis for *Gmcupins* during soybean domestication

Thirty-five *Gmcupin* genes were analyzed for the selection effects during soybean domestication based on the sequence diversity analysis between seventeen wild soybean and fourteen cultivars. The reverse distribution of SNPs in different evolutionary type of soybeans was defined as strong selected sites, and then *Cupin* genes with one or more type of reverse distribution were assumed to undergo an artificial selection during soybean domestication. Sixteen *Gmcupins* have selected site(s), among which more than one selected sites were determined in 8 *Gmcypins* and one selected sites in 8 genes (Table 3). Additionally, all SNP sites were selected in *Gmcupin10.3* and *Gmcupin07.2* genes, which implied these genes may have undergone strong selection effects during soybean domestication. Interestingly, selected sites were identified in *Gmcupin03.1* (7 sites), *Gmcupin13.2* (1 site) and *Gmcupin19.13* (1 site) that were highly expressed at the later stages of soybean seed. The genetic diversity of most *Gmcupins* was declined sharply in cultivars compared with that of wild soybeans. However, *Gmcupin10.7* gene that specifically expressed in soybean root showed three types of haplotype in wild soybeans, while four types of haplotype were identified in cultivars. Further, a new type of haplotype in *Gmcupin10.7* appeared during soybean domestication under the pressure of artificial selection, which would endow it with new functions. These selected genes reflected the important roles of *Gmcupins* on soybean domestication and contribute to the cultivation of soybeans in order to meet the demands of human beings.

**Table 3:** Selected sites of *Gmcupin* genes during soybean domestication.

Name	Position	SNPs		Name	Position	SNPs	
		Wild	Cultivar			Wild	Cultivar
Gmcupin03.1	39840871	11C/5T	5C/9T	Gmcupin10.3	36808227	9T/7A	1T/13A
	39841560	8G/7T	0G/13T		36808469	10G/5A	1G/13A
	39841777	8C/3T	3C/8T		36808488	10A/5G	1A/13G
	39842153	10T/6A	6T/8A		36808586	10A/4T	1A/13T
	39842186	9T/5C	4T/7C	Gmcupin10.4	36812510	9C/6T	1C/13T
	39842508	10A/5G	5A/9G		36812530	10G/6A	1G/13A
	39842704	10T/6A	5T/8A		36812729	8G/6C	1G/13C
Gmcupin07.1	3163748	12A/4C	4A/9C	Gmcupin10.7	39767387	14T/1C	4T/5C
	3164179	10A/5T	4A/9T		39768245	11T/1A	4T/6A
Gmcupin07.2	3167526	13T/4C	4T/10C	Gmcupin13.1	20816255	13C/4A	5C/8A
	3167819	12T/4C	4T/10C	Gmcupin13.2	22112820	9C/7G	5C/9G
	3167944	14G/3T	4G/10T	Gmcupin16.4	5853162	12C/4G	6C/8G
	3168029	13T/2C	4T/8C		5853378	11A/6G	6A/7G
	3168069	13T/2A	4T/8A		5853406	11G/5T	6G/8T
Gmcupin07.4	3179898	14T/2C	4T/10C	Gmcupin16.8	5951996	10T/4C	3T/7C
Gmcupin10.1	7200497	9C/6A	2C/12A	Gmcupin17.1	4052528	10A/5C	4A/9C
	7200888	13A/4G	2A/12G	Gmcupin19.13	42367147	10C/4T	6C/8T
	7200956	8G/8A	2G/12A	Gmcupin19.15	47524873	8A/8G	5A/9G
	7201734	12T/4C	2T/12C	Gmcupin20.1	32106107	9G/8T	1G/12T

## Supporting Information

Table S1 Pairwise identities between homologous pairs of Cupin genes from soybean.
(DOC)
