# Supplementary material for: A Comprehensive Analysis of the Cupin Gene Family in Soybean (Glycine max)
Source: PLoS One. 2014 Oct 31;9(10):e110092. doi: 10.1371/journal.pone.0110092 (PMC4215997; doi:10.1371/journal.pone.0110092)
Supplement: Table S1 — Pairwise identities between homologous pairs of Cupin genes from soybean. (DOC) [file pone.0110092.s001.doc]

Table S1. Pairwise identities between homologous pairs of Cupin genes from soybean

|  | **Bits** | **Identities** | **Similarities** | **Gaps** | **Fragment duplication** |
| --- | --- | --- | --- | --- | --- |
| Gmcupin19.8/19.10 | 1141 | 222/222(100.0%) | 222/222(100.0%) | 0/222(0.0%) | None |
| Gmcupin19.7/19.11 | 1043.5 | 208/222(93.7%) | 209/222(94.1%) | 11/222(5.0%) | None |
| Gmcupin16.8/19.12 | 745 | 141/212(66.5%) | 172/212(81.1%) | 0/212(0.0%) | Small |
| Gmcupin10.6/20.4 | 921.5 | 182/223(81.6%) | 189/223(84.8%) | 24/223(10.8%) | Large |
| Gmcupin10.7/20.3 | 1155 | 219/233(94.0%) | 226/233(97.0%) | 0/233(0.0%) | Large |
| Gmcupin05.1/08.1 | 1044 | 199/216(92.1%) | 208/216(96.3%) | 0/216(0.0%) | Large |
| Gmcupin09.1/15.2 | 1020 | 202/218(92.7%) | 205/218(94.0%) | 2/218(0.9%) | Large |
| Gmcupin10.3/10.4 | 1037 | 205/222(92.3%) | 209/222(94.1%) | 1/222(0.5%) | Tandem duplication |
| Gmcupin03.2/19.15 | 1045 | 209/220(95.0%) | 215/220(97.7%) | 1/220(0.5%) | Large |
| Gmcupin10.5/20.1 | 1081 | 215/224(96.0%) | 219/224(97.8%) | 0/224(0.0%) | Small |
| Gmcupin01.1/02.2 | 1107 | 214/221(96.8%) | 218/221(98.6%) | 0/221(0.0%) | Large |
| Gmcupin12.4/20.2 | 1023 | 201/208(96.6%) | 204/208(98.1%) | 0/208(0.0%) | None |
| Gmcupin12.1/12.3 | 1045 | 208/208(100.0%) | 208/208(100.0%) | 0/208(0.0%) | None |
| Gmcupin10.1/13.3 | 1070 | 206/227(90.7%) | 215/227(94.7%) | 0/227(0.0%) | Large |
| Gmcupin04.1/06.1 | 754.5 | 155/215(72.1%) | 166/215(77.2%) | 34/215(15.8%) | Large |
| Gmcupin08.2/15.4 | 1017 | 198/211(93.8%) | 202/211(95.7%) | 0/211(0.0%) | Small |
| Gmcupin13.1/17.1 | 745 | 157/189(83.1%) | 161/189(85.2%) | 16/189(8.5%) | Small |
| Gmcupin07.1/16.1 | 1029 | 203/210(96.7%) | 206/210(98.1%) | 0/210(0.0%) | Large |
| Gmcupin16.10/16.11 | 942 | 180/183(98.4%) | 182/183(99.5%) | 0/183(0.0%) | None |
| Gmcupin07.3/07.5 | 1073 | 209/209(100.0%) | 209/209(100.0%) | 0/209(0.0%) | None |
| Gmcupin13.4/15.1 | 529.5 | 107/144(74.3%) | 117/144(81.2%) | 19/144(13.2%) | Large |
| Gmcupin03.1/19.13 | 2229.5 | 428/498(85.9%) | 453/498(91.0%) | 18/498(3.6%) | Large |
